# Multiplication of Dentists in the West

**Published:** 1853-10

**Authors:** 


*Multiplication of Dentists in the West.*—An esteemed correspondent, writing from Illinois, says: "Dentists are multiplying in the west, still I know but few who are well qualified. Some have art without science, others have science without art; some have neither, and all think themselves dentists. The valley of the Mississippi, is a great field. The laborers are few. I hope you will send us more, such as are thoroughly furnished for the work." He also adds, that there are many good towns there in which capable young practitioners could do well.

We thank our correspondent for the kind invitation, and the liberality of feeling which prompted him to give it; but as yet, we have none such as he writes for, to spare. We have not enough at this time to supply the demand east of the mountains; but, we will do all we can to meet his wishes, and have no doubt, our friends a little further east and north, as well as those of the Queen City, will aid us in our charitable desire to oblige him.

Twenty-five or thirty years ago, there were not more than a hundred dentists in the United States; now, there are about four thousand, and still the demand for the services of practitioners of dentistry, cannot be as easily supplied now as it was then. Where one person employed a dentist then, a hundred do it now. Fifteen thousand dollars worth of gold foil, was, probably, as much as was used by all the dentists in the United States in a year, at that time. Now, two hundred thousand dollars worth scarcely supplies the demand. Should the increase in the quantity used, continue in the same ratio for the nəxt twenty-five or thirty years, the amount then, annually employed, would be enormous; it would exceed considerably two million dollars, and that it will thus continue to increase, there is not the slightest doubt.

